# ER-Poor and HER2-Positive: A Potential Subtype of Breast Cancer to Avoid Axillary Dissection in Node Positive Patients after Neoadjuvant Chemo-Trastuzumab Therapy

**DOI:** 10.1371/journal.pone.0114646

**Published:** 2014-12-11

**Authors:** Jian-wei Li, Miao Mo, Ke-da Yu, Can-ming Chen, Zhen Hu, Yi-feng Hou, Gen-hong Di, Jiong Wu, Zhen-zhou Shen, Zhi-ming Shao, Guang-yu Liu

**Affiliations:** 1 Department of Breast Surgery, Shanghai Cancer Center/Cancer Institute, Fudan University, Shanghai, P. R. China and Department of Oncology, Shanghai Medical College, Fudan University, Shanghai, P. R. China; 2 Clinical Statistics Center, Fudan University Shanghai Cancer Center, Shanghai, P. R. China and Department of Oncology, Shanghai Medical College, Fudan University, Shanghai, P. R. China; Johns Hopkins University, United States of America

## Abstract

**Purpose:**

The study was to estimate the likelihood of axillary downstaging and to identify the factors predicting a pathologically node negative status after neoadjuvant chemotherapy (NAC) with or without trastuzumab in HER2-positive breast cancer.

**Methods:**

Patients with HER2-positive, stage IIa-IIIc breast cancer were enrolled. Axillary status was evaluated by palpation and fine needle aspiration (FNA) before NAC. All patients received 4–6 cycles of PCrb (paclitaxel 80 mg/m^2^ and carboplatin AUC = 2 d1, 8, and 15 of a 28-day cycle, or paclitaxel 175 mg/m^2^ and carboplatin AUC = 6 every-3-week) and were non-randomly administered trastuzumab (2 mg/kg weekly or 6 mg/kg every-3-week) or not. After NAC, each patient underwent standard axillary lymph node dissection and breast-conserving surgery or mastectomy. And some patients received sentinel lymph node biopsy (SLNB) before axillary dissection.

**Results:**

Between November-2007 and June-2013, 255 patients were enrolled. Of them, 157 were confirmed as axillary node positive by FNA (group-A) and 98 as axillary node negative either by FNA or impalpable (group-B). After axillary dissection, the overall pathologically node negative rates (pNNR) were 52.9% in group-A and 69.4% in group-B. The ER-poor/HER2-positive subtype acquired the highest pNNR (79.6% in group-A and 87.9% in group-B, respectively) and the lowest rate of residual with ≥4 nodes involvement (1.9% and 3%, respectively) after PCrb plus trastuzumab. In multivariate analysis, trastuzumab added and ER-poor status were independent factors in predicting a higher pNNR in HER2-positive breast cancer. Forty-six tested patients showed that the ER-poor/HER2-positive subtype acquired a considerable high pNNR and axillary status with SLNB was well macthed with the axillary dissection.

**Conclusions:**

ER-poor/HER2-positive subtype of breast cancer is a potential candidate for undergoing sentinel lymph node biopsy instead of regional node dissection for accurate axillary evaluation after effective downstaging by neoadjuvant chemo-trastuzumab therapy.

## Introduction

Although sentinel lymph node biopsy (SLNB) is a good substitute for axillary dissection in some situations, axillary dissection currently remains the standard of care for axillary node positive operable breast cancer [1]. However, axillary dissection would significantly increase the risk of severe morbidity of the ipsilateral arm, such as lymphatic edema, pain and dyskinesia [2], [3].

Neoadjuvant chemotherapy (NAC) has become a standard treatment for locally advanced breast cancer to downstage tumors, which aids in surgical resection [4]. Additionally, downsizing the primary tumor can make breast-conservative surgery (BCS) possible for some patients who were initial candidates for mastectomy and therefore improve cosmetic outcomes [5], [6]. Furthermore, patients who achieved pathologic complete remission (pCR) after NAC would acquire better long-term survival than those who did not. The pCR occurs not only in the primary tumor but also in the involved axillary nodes [7]. Therefore, axillary dissection might be avoided or replaced by a more conservative procedure in a certain subtype of node positive breast cancer patients after NAC.

Amplification of human epidermal growth factor receptor-2 (HER2) is identified in approximately 20–25% of all human breast cancers [8]. Many studies have reported that breast cancers with HER2-positive can achieve a high pCR rate after NAC, especially using regimens including trastuzumab [9], [10]. However, the data about predicting axilla downstaging and pathologically node negative status after NAC are rare. The aim of our current study was to identify the likelihood of axillary downstaging and to seek the factors that may predict a pathologically node negative status after NAC in HER2-positive breast cancer.

## Patients and Methods

### Patients Information

A retrospective analysis was carried out among patients from the phase II trial evaluating the activity and safety of a weekly paclitaxel plus carboplatin (PCrb) regimen as NAC in women with locally advanced breast cancer (ClinicalTrials.gov NCT01203267) [11] and the two-arm randomized phase II trial comparing the efficacy and safety between weekly scheduled PCrb with weekly herceptine and once-every-3-week schedule in women with HER2-positive aggressive breast cancer (ClinicalTrials.gov NCT01170143) [12]. Untreated patients at ages of 18–80 years old with primary histologically confirmed, clinical stage IIa-IIIc, HER2-positive, invasive breast cancer were included. Patients with distant metastasis or prior history of malignancy were excluded. Up to June 2013, 255 patients were included for main analysis. And another 46 newly diagnosed patients were enrolled to identify and confirm the previous results. They were all with Eastern Cooperative Oncology Group performance status ≤1 and left ventricular ejection fraction>55% by multiple gated acquisition scan or echocardiography. Besides, they all had adequate hematopoietic function (absolute neutrophil count ≥1.5*10^9^/L, platelet count ≥100*10^9^/L, and hemoglobin level ≥100 g/L) and appreciate hepatic and renal function (bilirubin level, aspartate aminotransferase, and alanine aminotransferase <1.5 times the normal upper limit and serum creatinine <110 umol/L). Women of childbearing potential were required to have a negative pregnancy test and to agree to take adequate contraceptive measures.Immunohistochemical (IHC) assessment of estrogen receptor (ER), progesterone receptor (PR) and HER2 expression were conducted in paraffin-embedded tumor samples biopsied before neoadjuvant treatment according to the guidelines from the American Society of Clinical Oncology and the College of American Pathologists. The intensity was graded as 0 (poor), 1 (weak), 2 (moderate) or 3 (strong). The abundance of positive cells was graded as 0, <10% positive cells; 1, 10–30%; 2, 30–50%; 3,>50%; the scoring system of IHC for ER and PR is automated. All of the immunostained slides were analyzed independently by two pathologists without any knowledge of the clinicopathological features. HER2 positivity was determined by IHC 3 (HerceptTest; Dako, Glostrup, Copenhagen, Denmark, http://www.dako.com) or fluorescence *in situ* hybridization (FISH) positive status (PathVysion HER-2 DNA probekit; Vysis Inc., Downers Grove, IL) [13]–[15].

This study was approved by the Ethical Committee of Shanghai Cancer Center, Fudan University. Every patient signed a written informed consent.

### Procedures

**Figure 1:**
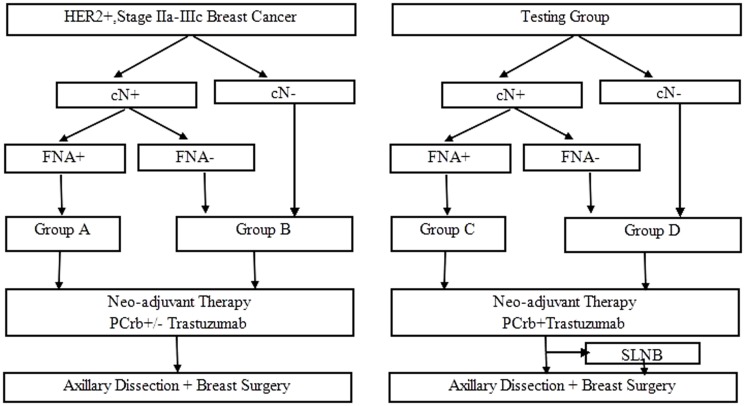
The retrospective study and testing group designs. HER2 =  human epideminal growth factor receptor 2; cN+ =  clinically axillary node positive; cN- =  clinically axillary node negative; FNA =  fine needle aspiration; P =  paclitaxel; Crb =  carboplatin; SLNB =  sentinel lymph node biopsy. Some patients received SLNB before axillary dissection in the testing group for exploring the accuracy of SLNB after neoadjuvant therapy.

We sorted patients to two study groups for both 255 and 46 patients according to the baseline axillary nodal status, determined by both palpation and fine-needle aspiration (FNA), before NAC (Fig. 1). Group-A and C included patients with an axillary node positive status confirmed by FNA (cN+FNA+). Group-B and D included patients with clinically enlarged axillary node(s) that were negative by FNA (cN+/FNA-) or patients with clinically impalpable node (cN-). We classified tumor deposits according to the Union for International Cancer Control TNM classification system [16].

For main analysis, all patients were non-randomly administered PCrbH (weekly or every-3-week) or PCrb (weekly) for 4–6 cycles. In weekly schedule, patients received an 80 mg/m^2^ dose of paclitaxel (P) followed by carboplatin (Crb) dosed at an area under the concentration-time curve (AUC) of 2 on days 1, 8, and 15 of a 28-day cycle with or without trastuzumab given at a dose of 2 mg/kg (with a loading dose of 4 mg/kg) every week. In every-3-week schedule, patients received 175 mg/m^2^ paclitaxel followed by carboplatin at an AUC of 6 on day 1 of a 21-day cycle with or without trastuzumab at 6 mg/kg (with a loading dose of 8 mg/kg) every 3 weeks. 46 newly diagnosed patients all administered PCrbH (weekly or every-3-week) for 4-6 cycles. Breast surgery (BCS or mastectomy) and standard axillary dissection were performed 2–4 weeks after the last chemotherapy dose. 18 of 46 patients received SLNB just before axillary dissection. The surgery type was at the surgeon's discretion.

The primary end point was to evaluate the factors influencing the pathologically node negative rate (pNNR) of the axillary lymph nodes, which was defined as the absence of malignant cells in all lymph nodes removed by axillary dissection assessed microscopically with hematoxylin/eosin staining.

### Statistical Analysis

Medians and ranges of demographic characteristics and distribution of clinical characteristics were presented. Clinical characteristics between pNNR and non-pNNR subgroups for both Group-A and Group-B patients were compared using Pearson's χ^2^test or Fisher's exact test where appropriate. Similar analyses were done between Group-C and Group-D patients to identify and confirm the previous results. Multivariate analysis was performed using logistic regression analysis. The regimen, menopausal status, baseline tumor stage and ER/PR status were taken into account in the multivariate analysis. The hazard ratios (HRs) with 95% confidence intervals (CIs) were calculated. All tests were two-sided and *P*-value less than 0.05 was considered statistically significant. All statistical analyses were performed using SPSS v.19.0 (SPSS, Chicago, IL).

## Results

Between November-2007 and June-2013, 255 patients with HER2-positive breast cancer were enrolled, with median age of 50 years. One hundred and fifty-seven 157(61.6%) patients with cN+FNA+ were included in group-A and 98 (38.4%) patients with cN+FNA- (n = 64) or cN- (n = 34) were included in group-B. The patients' major characteristics were shown in Table 1. The overall pCR rate of the primary site was 42.5%, with 39.5% in group-A and 45.9% in group-B.

**Table 1:** The demographic characteristics and clinicopathological information of 255 patients for the main analysis.

Characteristics (n, %)	Group-A,cN+FNA+ N = 157	Group-B,cN+FNA- N = 64	Group-B,cN- N = 34	Total N = 255
Median age* yr(range)	51(21–71)	49.5(26–79)	51.5(27–63)	50(21–79)
NAC regimen				
PCrbH	95(60.5%)	39(60.9%)	15(44.1%)	149(58.4%)
PCrb	62(39.5%)	25(39.1%)	19(55.9%)	106(41.6%)
Median BSA, m^2^(range)	1.60(1.31–1.96)	1.58(1.37–2.00)	1.58(1.40–2.26)	1.60(1.31–2.26)
Median BMI(range)	23.2(19.9–32.1)	23.2(15.9–33.5)	23.7(16.9–40.4)	23.2(15.9–23.7)
Menopausal status				
Pre-	82(52.2%)	32(50.0%)	21(61.8%)	135(52.9%)
Post-	75(47.8%)	32(50.0%)	13(38.2%)	120(47.1%)
T				
1	10(6.4%)	2(3.1%)	1(2.9%)	13(5.1%)
2	49(31.2%)	20(31.3%)	13(38.2%)	82(32.2%)
3	22(14.0%)	12(18.8%)	7(20.6%)	41(16.1%)
4	76(48.4%)	30(46.9%)	13(38.2%)	119(46.7%)
N				
0	0(0.0%)	56(87.5%)	0(0.0%)	56(22.0%)
1	117(74.5%)	0(0.0%)	0(0.0%)	117(45.6%)
2	14(8.9%)	0(0.0%)	0(0.0%)	14(5.5%)
3	26(16.6%)	8(12.5%)	4(11.8%)	38(14.9%)
unknown	0(0.0%)	0(0.0%)	30(88.2%)	30(11.8%)
Estrogen receptor				
Positive	67(42.7%)	20(31.3%)	16(47.1%)	103(40.4%)
Poor	90(57.3%)	44(68.7%)	18(52.9%)	152(59.6%)
Progesterone receptor				
Positive	66(42.0%)	21(32.8%)	18(52.9%)	105(41.2%)
Poor	91(58.0%)	43(67.2%)	16(47.1%)	150(58.8%)
Breast Surgery				
Mastectomy	147(93.6%)	62(96.9%)	34(100%)	243(95.3%)
BCS	10(6.4%)	2(3.1%)	0(0.0%)	12(4.7%)

Data was given as the number and percent (n/N, %) of patients, unless otherwise stated.

*Median and full range.

FNA =  fine-needle aspiration; NAC =  neoadjuvant chemotherapy; P =  paclitaxel; Crb =  carboplatin; H =  Trastuzumab (Herceptin); T =  primary tumor site of TNM classification system; N =  the regional lymph node involvement of TNM classification system; BCS =  breast conserving surgery; ALND =  axillary lymph node dissection.

**Figure 2:**
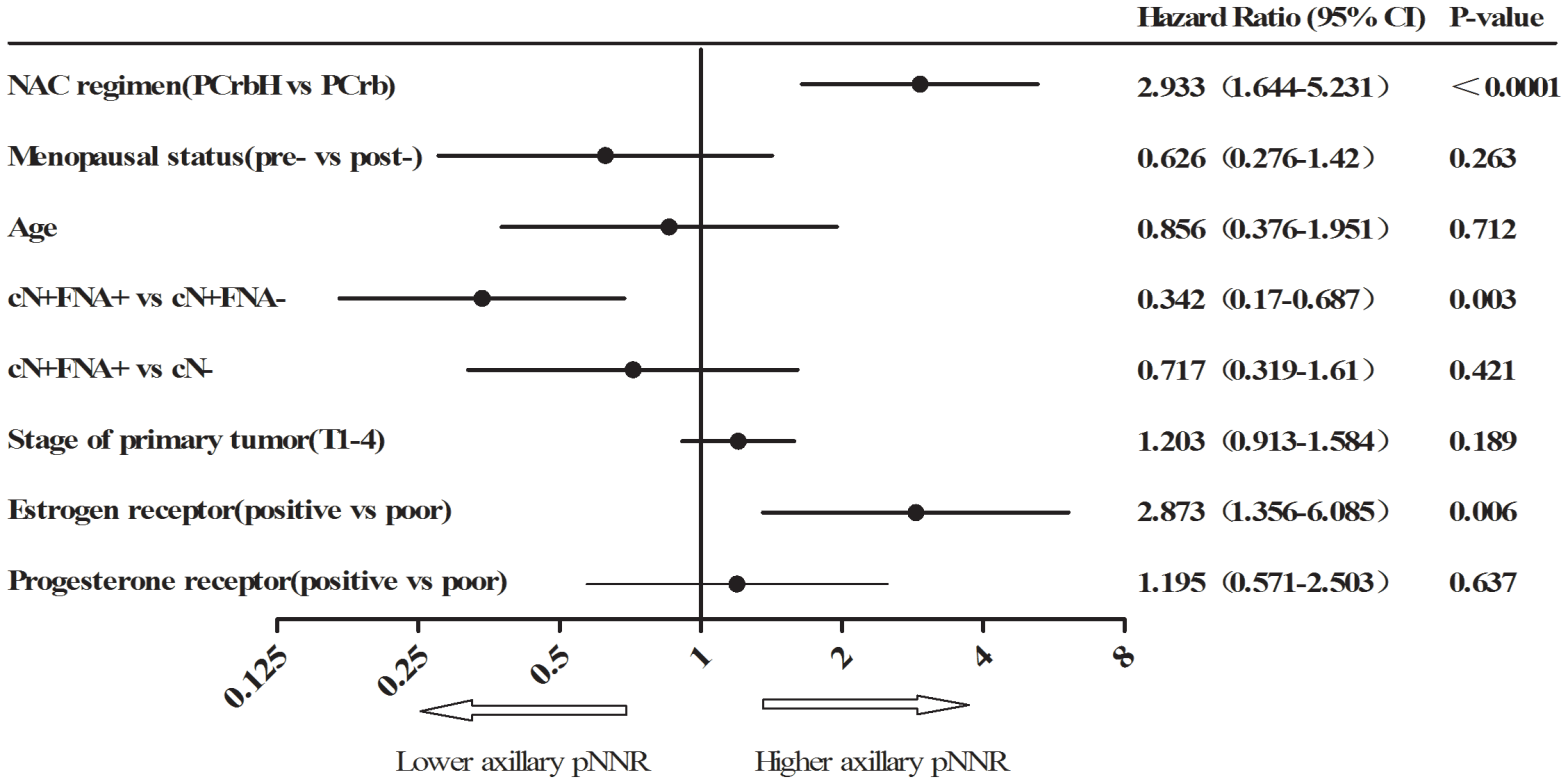
Multivariate regression analysis for axillary pNNR. NAC =  neoadjuvant chemotherapy; pNNR =  pathologically node negative rate; P =  paclitaxel; Crb =  carboplatin; H =  Trastuzumab (Herceptin). In multivariate analysis, trastuzumab added and ER-poor status showed higher pNNR in HER2-positive breast cancer.

After axillary dissection, the overall pNNR were 52.7% and 69.4% in group-A and group-B, respectively. Axillary pNNR was highly correlated with the pCR rate of the primary site (*p*<0.0001). With the PCrbH regimen, 61.6% patients achieved pNNR in group-A and 81.5% in group-B, which were significantly higher than those treated with the PCrb regimen (*p* = 0.011 in group-A and *p* = 0.004 in group-B). The outcomes of axillary dissection in different patient subgroups were listed in Table 2. The subtype of ER-poor and HER2-positive breast cancer treated with neoadjuvant PCrbH showed the highest pNNR (79.6% in group-A and 87.9% in group-B) and the least residual (≥4 nodes) involvement (1.9% in group-A and 3% in group-B). These findings were also confirmed by multivariate analysis which indicated that trastuzumab addition to NAC (HR = 2.933) and an ER-poor status (HR = 2.873) may be related factors for predicting a higher pNNR after NAC in HER2-positive breast cancer (Fig. 2).

**Table 2:** Axillary nodal status after neo-adjuvant therapy according to different subgroups.

	Group-A (cN+/FNA+) N = 157				Group-B (cN+/FNA- or cN-) N = 98			
	pNNR	non-pNNR			pNNR	non-pNNR		
No. of involved nodes (n, %)	0	1–3	≥4	*P* value	0	1–3	≥4	*P* value
Overall	83(52.9%)	43(27.4%)	31(19.7%)		68(69.4%)	15(15.3%)	15(15.3%)	
NAC regimen				*0.011*				*0.004*
PCrbH	58(61.1%)	23(24.2%)	14(14.7%)		44(81.5%)	6(11.1%)	4(7.4%)	
PCrb	25(40.3%)	20(32.3%)	17(27.4%)		24(54.5%)	9(20.5%)	11(25%)	
Menopausal Status				*0.597*				*0.328*
Pre-	38(50.7%)	25(33.3%)	12(16.0%)		29(64.4%)	9(20.0%)	7(15.6%)	
Post-	45(54.9%)	18(22%)	19(23.2%)		39(73.6%)	6(11.3%)	8(15.1%)	
T stage				*0.661*				*0.418*
1	6(60.0%)	2(20.0%)	2(20.0%)		3(100.0%)	0(0.0%)	0(0.0%)	
2	29(59.2%)	9(18.4%)	11(22.4%)		20(60.6%)	7(21.2%)	6(18.2%)	
3	11(50.0%)	7(31.8%)	4(18.2%)		14(73.7%)	3(15.8%)	2(10.5%)	
4	37(48.7%)	25(32.9%)	14(18.4%)		31(72.1%)	5(11.6%)	7(16.3%)	
ER				*0.002*				*0.024*
Positive	26(38.8%)	19(28.4%)	22(32.8%)		20(55.6%)	7(19.4%)	9(25.0%)	
Poor	57(63.3%)	24(26.7%)	9(10.0%)		48(77.4%)	8(12.9%)	6(9.7%)	
PgR				*0.113*				*0.007*
Positive	30(45.5%)	18(27.3%)	18(27.3%)		21(53.8%)	8(20.5%)	10(25.6%)	
Poor	53(58.2%)	25(27.5%)	13(14.3%)		47(79.7%)	7(11.9%)	5(8.5%)	
NAC regimen & ER status								
PCrbH				*<0.0001*				*0.129*
ER Positive	15(36.6%)	13(31.7%)	13(31.7%)		15(71.4%)	3(14.3%)	3(14.3%)	
ER-poor	**43(79.6%)**	**10(18.5%)**	**1(1.9%)**		**29(87.9%)**	**3(9.1%)**	**1(3.0%)**	
PCrb				*0.787*				*0.042*
ER Positive	11(42.3%)	6(23.1%)	9(34.6%)		5(33.3%)	4(26.7%)	6(40.0%)	
ER-poor	14(38.9%)	14(38.9%)	8(22.2%)		19(65.5%)	5(17.2%)	5(17.2%)	

Data was given as the number and percent (n/N, %) of patients.

FNA =  fine needle aspiration; pNNR =  pathologically node negative rate; NAC =  neoadjuvant chemotherapy; P =  paclitaxel; Crb =  carboplatin; H =  Trastuzumab (Herceptin); ER =  estrogen receptor; PgR =  progesterone receptor; T =  primary tumor site of TNM classification system.

*P* values were calculated from χ^2^ test to compare pNNR and non-pNNR proportions of patients for different subgroups.

From June 2013 to March 2014, another 46 patients were enrolled to identify and confirm the previous results, 36 (78.3%) with positive axillary nodes (group-C) and 10 (21.7%) negative both by FNA (group-D). The patients' major characteristics were similar with the main study set. They all had a NAC regimen of PCrbH. After axillary dissection, the overall pNNR was 61.1% and 60.0% for group-C and group-D, respectively. The ER-poor/HER2-positive subtype acquired considerable high pNNRs (77.8% in group-C and 71.4% in group-D, seen in S1 Table).

Among 18 patients received SLNB before axillary dissection, SLN was successfully detected in 16 (88.9%). As shown in S2 Table, all 5 ER-poor patients with negative SLN showed pathologically negative node (100%) after axillary dissection.

## Discussion

Immunohistochemical phenotype of ER-poor and HER2-positive breast cancer accounts for approximately 10-15% of early breast cancers. They have been found more sensitive to chemo-trastuzumab therapy in the neoadjuvant setting with a higher pCR rate in the primary site than that of the ER-positive/HER2-positive phenotype, as shown in Table 3 [9], [17]–[20]. In our current study, we observed optimal pathological axillary node downstaging of HER2-positive breast cancer after an NAC regimen of PCrbH. This result also indicated that the therapeutic sensitivity of breast cancer metastasis to the axilla is at least equal to or even higher than that of the primary site.

**Table 3:** pCR rate after neoadjuvant chemotherapy plus trastuzumab in breast cancer.

Studies	Phase	Clinical stage	Neoadjuvant regimen	pCR (%)	pCR(%) for HR-positive	pCR(%) for HR-negative
MD Anderson [9]	III	II–III	P+T→FEC+T	65.0	61.5	70.0
NOAH [17]	III	III	AP+T→P+T→CMF+T	38.0	18.0	38.0
NeoALTTO [18]	III	II–III	Weekly P+T	28.0	22.7	36.5
NeoSphere [19]	III	II–III	D+T	23.0	20.0	36.8
NSABP B-41 [20]	III	II–III	AC→weekly P+T	49.4	46.7	65.5

A =  doxorubicin; CMF =  cyclophosphamide, methotrexate and 5- fluorouracil; D =  docetaxel; FEC =  5-fluorouracil, epirubicin and cyclophosphamide; P =  paclitaxel; T =  trastuzumab.

pCR was defined as no invasive tumor in the breast and axilla.

For clinically node negative early breast cancer, SLNB is now routinely used as a standard method for pathological staging of the axilla. SLNB has little morbidity and high accuracy that is comparable with traditional axillary dissection [21]. However, the limitations of SLNB for evaluating a downstaged breast cancer after NAC should be considered, because of the increased false negative rate (FNR: 5.6–35.5%) and decreased detection rate (77.6–98.0%) [22]–[26]. SENTINA (SENTinel NeoAdjuvant) trial is thus far the largest prospective, multicenter cohort study to investigate the feasibility and accuracy of SLNB after NAC [27]. In this study, 592 breast cancer patients who converted from a clinically axillary node positive to negative status after NAC received SLNB just before axillary dissection. A relatively lower sentinel node detection rate of 80.1% (compared with 99.1% in SLNB before NAC) and a FNR of 14.2% were observed. The ACOSOG Z1071 trial was designed to determine the FNR of SLN surgery after chemotherapy in women initially presenting with cN1 disease [28]. In this trial, the sentinel node detection (≥2 SLN) rate was 80.9% and the FNR was 12.6%. According to these reports, the missing rate of SLNB after NAC far exceeded the acceptable extension of <5% recommended by the ASCO guideline [29]. However, if there was a subgroup of patients who were more likely to have a pathologically node negative status in the axilla after NAC, the false negative error from SLNB would decrease and most of the axillary dissection could be avoided for them. For example, in group-A from our study, a subgroup (n = 54) of FNA confirmed node positive patients with the ER-poor and HER2-positive phenotype achieved a pNNR of 79.6% after neoadjuvant PCrbH. Supposing that these tumors had the same FNR and detection rate of SLNB as reported in Z1071 trial [28], axillary dissection could safely be avoided in approximately two thirds of the patients from a successful and true negative SLNB (pNNR×Detection Rate [79.6%*80.9%] = 64.40%) with an estimated acceptable false negative error rate of about 2 cases per 100 patients ([1-pNNR]×FNR×Detection Rate = 2.08%).

For patients with clinically node negative disease who require NAC (i.e patients in group-B and group-D of the study), the timing of SLNB is still controversial. Conducting SLNB before NAC can provide an accurate histological evaluation of the initial axillary nodal status, which might be essential in the prognosis estimation and for guidance of the following loco regional treatment, including axillary dissection and radiation. However, growing evidences have suggested that the nodal status after NAC reflects the prognosis more accurately than the initial axillary status [30]. Thus, a reliable estimation of an excellent regional node response to the NAC could also change the current strategy of axillary dissection to a more conservative approach. Unfortunately, according to the data from the SENTINA trial, if SLNB was performed before NAC and identified as positive, a second SLNB after NAC was far less reliable than axillary dissection, with a detection rate of only 60.8% and a FNR of 51.6% [27]. For example, in group-B of the current study, a subgroup (n = 33) of clinically node negative patients with the ER-poor and HER2-positive phenotype achieved the highest pNNR (87.9%) after neoadjuvant PCrbH. According to the results from the SENTINA trial, if SLNB is scheduled before NAC, the repeat SLNB for those sentinel node positive patients after NAC might result in a false negative error rate of 3.79%. Alternatively, a single procedure of SLNB scheduled after NAC could be more feasible for this group of patients and the axillary dissection could be safely avoided in at least 70% of the patients with a lower false negative error rate that is expected to be 1.38%.

Recently, data from some prospective studies indicated that if there is not palpable adenopathy breast cancer with positive axillary sentinel nodes (micro-metastatic size<2 mm or 1–3 macro-metastatic nodes) has no significant difference in regional recurrence rate, disease free survival or overall survival for complete axillary dissection versus axillary nodal irradiation or observation except that the complications were reduced in the axilla conservation groups [31]–[33]. In the current study, very few patients (1.9% in group-A and 3% in group-B) with the ER-poor and HER2-positive phenotype had more than 3 macro-metastatic nodes remaining in the axilla after neoadjuvant PCrbH. Therefore, with the replacement of axillary irradiation, avoidance of axillary dissection in the ER-poor and HER2-positive subtype might be extended to patients with 1–3 positive sentinel nodes including those who failed to achieve a successful SLNB after NAC. Further studies are needed to verify these results.

## Conclusions

In conclusion, patients with ER-poor and HER2-positive subtype of breast cancer are potential candidates for avoiding unnecessary axillary dissection due to its higher axillary node negative status rate after neoadjuvant chemo-trastuzumab therapy. Further prospective trials should be focused on SLNB and axillary irradiation after neoadjuvant chemo-trastuzumab therapy to take the place of traditional axillary dissection in the ER-poor and HER2-positive subtype of breast cancer.

## Supporting Information

S1 Table **Axillary nodal status after neo-adjuvant therapy according to ER status among 46 newly patients.** Data was given as the number and percent (n/N, %) of patients. FNA =  fine needle aspiration; pNNR =  pathologically node negative rate; ER = estrogen receptor.
(DOC)

S2 Table **Axillary nodal status after neo-adjuvant therapy according to ER status among patients received sentinel lymph node biopsy before axillary dissection.** Data was given as the number and percent (n/N, %) of patients. SLNB =  sentinel lymph node biopsy; pNNR =  pathologically node negative rate; ER = estrogen receptor.
(DOC)
