# Supplementary material for: ER-Poor and HER2-Positive: A Potential Subtype of Breast Cancer to Avoid Axillary Dissection in Node Positive Patients after Neoadjuvant Chemo-Trastuzumab Therapy
Source: PLoS One. 2014 Dec 11;9(12):e114646. doi: 10.1371/journal.pone.0114646 (PMC4263615; doi:10.1371/journal.pone.0114646)
Supplement: S1 Table — Axillary nodal status after neo-adjuvant therapy according to ER status among 46 newly patients. Data was given as the number and percent (n/N, %) of patients. FNA = fine needle aspiration; pNNR = pathologically node negative rate; ER = estrogen receptor. (DOC) [file pone.0114646.s001.doc]

**Table S1. Axillary nodal status after neo-adjuvant therapy according to ER status among 46 newly patients**

|  | Group-C (FNA+)  N=36 | | | | | Group-D (FNA-)  N=10 | | | |
| --- | --- | --- | --- | --- | --- | --- | --- | --- | --- |
|  | pNNR | non-pNNR | |  | pNNR | | non-pNNR | |  |
| No. of involved nodes (n, %) | 0 | 1-3 | ≥4 | *P value* | 0 | | 1-3 | ≥4 | *P value* |
| Overall | 22(61.1%) | 7(19.4%) | 7(19.4%) |  | 6(60.0%) | | 4(40.0%) | 0(0%) |  |
| ER |  |  |  | *0.040* |  | |  |  | *0.260* |
| Positive | 8(44.4%) | 5(27.8%) | 5(27.8%) |  | 1(33.3%) | | 2(66.7%) | 0(0%) |  |
| Poor | **14(77.8%)** | **2(11.1%)** | **2(11.1%)** |  | **5(71.4%)** | | **2(28.6%)** | **0(0%)** |  |

Data was given as the number and percent (n/N, %) of patients.

FNA = fine needle aspiration; pNNR = pathologically node negative rate; ER=estrogen receptor.
