# Supplementary material for: ER-Poor and HER2-Positive: A Potential Subtype of Breast Cancer to Avoid Axillary Dissection in Node Positive Patients after Neoadjuvant Chemo-Trastuzumab Therapy
Source: PLoS One. 2014 Dec 11;9(12):e114646. doi: 10.1371/journal.pone.0114646 (PMC4263615; doi:10.1371/journal.pone.0114646)
Supplement: S2 Table — Axillary nodal status after neo-adjuvant therapy according to ER status among patients received sentinel lymph node biopsy before axillary dissection. Data was given as the number and percent (n/N, %) of patients. SLNB = sentinel lymph node biopsy; pNNR = pathologically node negative rate; ER = estrogen receptor. (DOC) [file pone.0114646.s002.doc]

**Table S2. Axillary nodal status after neo-adjuvant therapy according to ER status among patients received sentinel lymph node biopsy before axillary dissection**

|  | SLNB+ (N=8） | | | | | SLNB- (N=8) | | | |
| --- | --- | --- | --- | --- | --- | --- | --- | --- | --- |
|  | pNNR | non-pNNR | |  | pNNR | | non-pNNR | |  |
| No. of involved nodes (n, %) | 0 | 1-3 | ≥4 |  | 0 | | 1-3 | ≥4 |  |
| Overall | 3(37.5%) | 3(37.5%) | 2(25.0%) |  | 6(75.0%) | | 2(25.0%) | 0(0%) |  |
| ER |  |  |  |  |  | |  |  |  |
| Positive | 1(50.0%) | 1(50.0%) | 0(0%) |  | 1(33.3%) | | 2(66.7%) | 0(0%) |  |
| Poor | 2(33.3%) | 2(33.3%) | 2(33.3%) |  | **5(100.0%)** | | **0(0%)** | **0(0%)** |  |

Data was given as the number and percent (n/N, %) of patients.

SLNB = sentinel lymph node biopsy; pNNR = pathologically node negative rate; ER=estrogen receptor.
